# Immune protection induced by E2 recombinant glycoprotein of bovine viral diarrhea virus in a murine model

**DOI:** 10.3389/fvets.2023.1168846

**Published:** 2023-06-22

**Authors:** Ninnet Gómez-Romero, Carlos F. Arias, Antonio Verdugo-Rodríguez, Susana López, Luis Fernando Valenzuela-Moreno, Carlos Cedillo-Peláez, Francisco Javier Basurto-Alcántara

**Affiliations:** ^1^Vaccinology Laboratory, Department of Microbiology and Immunology, Facultad de Medicina Veterinaria y Zootecnia-Universidad Nacional Autónoma de México, Mexico City, Mexico; ^2^Departamento de Genética del Desarrollo y Fisiología Molecular, Instituto de Biotecnología, Universidad Nacional Autónoma de México, Cuernavaca, Morelos, Mexico; ^3^Molecular Microbiology Laboratory, Department of Microbiology and Immunology, Facultad de Medicina Veterinaria y Zootecnia-Universidad Nacional Autónoma de México, Mexico City, Mexico; ^4^Laboratorio de Inmunología Experimental, Instituto Nacional de Pediatría, Mexico City, Mexico

**Keywords:** bovine viral diarrhea virus, E2 recombinant glycoprotein, murine model, adjuvant, virus neutralization

## Abstract

Bovine viral diarrhea virus (BVDV) is considered the most important viral pathogen in ruminants worldwide due to the broad range of clinical manifestations displayed by infected animals. Therefore, infection with BVDV leads to severe economic losses in several countries' beef and dairy industries. Vaccination prevents reproductive failure and gastrointestinal and respiratory disorders caused by BVDV infection. However, considering their limitations, conventional vaccines such as live, attenuated, and killed viruses have been applied. Hence, different studies have described subunit vaccines as an effective and safe alternative for BVDV protection. Therefore, in this study, the ectodomain of E2 (E2e) glycoprotein from NADL BVDV strain was expressed in mammalian cells and used in two vaccine formulations to evaluate immunogenicity and protection against BVDV conferred in a murine model. Formulations consisted of solo E2e glycoprotein and E2e glycoprotein emulsified in adjuvant ISA 61 VG. Five groups of 6 mice of 6-to-8-week-old were immunized thrice on days 1, 15, and 30 by intraperitoneal injection with the mentioned formulations and controls. To evaluate the conferred protection against BVDV, mice were challenged six weeks after the third immunization. In addition, the humoral immune response was evaluated after vaccination and challenge. Mice groups inoculated with solo E2e and the E2e + ISA 61 VG displayed neutralizing titers; however, the E2 antibody titers in the E2e + ISA 61 VG group were significantly higher than the mice group immunized with the solo E2e glycoprotein. In addition, immunization using E2e + ISA 61 VG prevents animals from developing severe lesions in surveyed tissues. Moreover, this group acquired protection against the BVDV challenge, evidenced by a significant reduction of positive staining for BVDV antigen in the lungs, liver, and brain between the experimental groups. Our findings demonstrated that using E2e + ISA 61 VG induces greater BVDV protection by an early humoral response and reduced histopathological lesions and BVDV antigen detection in affected organs, indicating that E2e + ISA 61 VG subunit formulation can be considered as a putative vaccine candidate against BVDV. The efficacy and safety of this vaccine candidate in cattle requires further investigation.

## Introduction

Bovine viral diarrhea virus (BVDV) is an important pathogen in cattle associated with reproductive failures such as abortions, mummifications, stillbirths, and the birth of persistently infected animals (PI) and also respiratory and enteric disorders causing severe economic losses. BVDV is the causative agent of bovine viral disease (BVD), which is considered one of the world's most economically significant bovine diseases (1). BVDV is a single-stranded RNA virus belonging to the *Pestivirus* genus within the *Flaviviridae* family. Currently, the three former species of BVDV-1, BVDV-2, and HoBi-like viruses are now referred to as *Pestivirus A, Pestivirus B*, and *Pestivirus H*, respectively (2). Moreover, phylogenetic analyses have further segregated these three pestiviruses into subgenotypes in at least 21 subgenotypes within BVDV-1, four BVDV-2 subgenotypes (a-d), and four HoBi-like virus subgenotypes (a-d) (3). BVDV genetic diversity and its distribution have been demonstrated previously in several regions worldwide. BVDV epidemiological studies in Mexican cattle revealed that BVDV-1a, 1b, and 1c and BVDV-2a, with no evidence of HoBi-like pestiviruses, are the prevalent subgenotypes (4). In Mexico, inactivated/killed and modified live vaccines (MLV) against BVDV are licensed and mainly determined to prevent clinical signs and control BVDV infections. However, some disadvantages of using these immunogens have been previously described. The MLV vaccines can cause reproductive disorders such as fetal infection, immunosuppression, recombination with BVDV field strains, development of mucosal disease in persistently infected cattle, and BVD attributable to vaccine contamination (5–8). On the contrary, during the inactivation process of killed vaccines, the immunogenicity of the viral antigen is reduced; thus, formulation with adjuvants is necessary. Additionally, killed vaccines induce short-term immune responses; hence, booster doses of inactivated vaccines are required to confer a protective immune response (9, 10).

Recently, subunit vaccine candidates based on BVDV E2 glycoprotein have been studied; however, the E2 expression system, dosage, formulations, and target animal species used remain a matter of evaluation and discussion (11–14). The envelope E2 protein is the immunodominant BVDV structural glycoprotein containing strongly neutralizing and CD8^+^ T-cell epitopes (15, 16). Furthermore, neutralizing antibodies induced in infected and vaccinated animals are mainly directed at the E2 protein (15, 17). Therefore, owing to the immunological response elicited by using E2 protein as a subunit vaccine, this BVDV glycoprotein remains a major target for vaccine design.

In this study, we evaluated the protective efficacy of two formulations of a recombinant vaccine based on the E2 glycoprotein in mice. Previous studies have revealed that mice are susceptible to BVDV infection, making the murine model a suitable option for studying BVDV infection (18, 19). Additionally, recent research has shown that the efficacy of compounds with antiviral properties against BVDV has been tested using this model (20, 21). Moreover, comparable lesions between mice and bovines are reported (22–24).

One of the formulations evaluated in this study consisted of the E2 protein emulsified in Montanide? ISA (SEPPIC, Paris, France) for vaccine optimization. The Montanide ISA? is a water-in-oil (W/O) mineral-oil-based adjuvant used to enhance the immunogenicity of antigens and stimulate strong immune responses against several viral antigens applied in cattle and other species. Vaccines for veterinarians that contain Montanide ISA 61 VG have been proven to stimulate an accelerated cellular and humoral immune response. Moreover, using Montanide ISA 61 VG resulted in higher titers of antibodies and IFN-gamma, slow antibody decay, and long-term protection compared to other adjuvants (25–27). In this study, we assessed the BVDV protection conferred using two experimental recombinant BVDV E2 protein formulations in a murine model.

## Materials and methods

### Ethics statement

All experimental protocols for animal trials were approved by the Care and Use for Experimental Animals Sub-committee from the Faculty of Veterinary Medicine of the National Autonomous University of Mexico (FMVZ-UNAM, for the acronym in Spanish).

### BVDV strain and virus titer determination

The BVDV-1a VR-534 (ATCC, Manassas, VA, USA) was cultured for gene cloning and vaccine production and used as a challenge strain. The viral strain was replicated in MDBK cells using Dulbecco's Minimum Essential Medium (DMEM) (Gibco™, Thermo Fisher Scientific, Waltham, MA, USA) supplemented with 10% equine serum (ATCC, Manassas, VA, USA). Viral titration was performed on MDBK's cells in 96-well plates using quintuple 10-fold dilutions, and titer was calculated using the Reed and Muench method (28).

### Cloning and expression of BVDV E2 glycoprotein

The gene encoding E2 glycoprotein BVDV NADL strain (~1,030 bp) with the deleted transmembrane domain was amplified using previously reported primers by Donofrio et al. (29) (Table 1) and cloned into the pSecTag2/Hygro A^®^ plasmid (Invitrogen, Carlsbad, CA, USA) using *Hind*III and *Xho*I restriction enzymes (New England BioLabs Inc., Massachusetts, USA). The recombinant plasmid pSecTag2E2 construct was confirmed by sequencing to establish accuracy. After that, transfection into HEK293T using lipofectamine 3000^^®^^ reagent (Thermo Fisher Scientific, Waltham, MA, USA) was performed. The transfected cells were selected with hygromycin (200 ug/mL) (Thermo Fisher Scientific, Waltham, MA, USA). Then, the surviving cells were recovered in 125 cm^2^ flasks with 40 ml of Dulbecco's modified medium supplemented with 10% of FBS (Gibco™, Thermo Fisher Scientific, Waltham, MA, USA) and used to produce recombinant E2e protein. The cell supernatant was collected, and protein purification was performed using Ni-NTA beads (Qiagen, Alameda, CA, USA) according to the manufacturer's indications.

**Table 1:** Primers used in this study for E2 amplification and detection of BVDV in mice tissue samples.

**Primers used in this study**			
**Primer ID**	**Primer sequence**	**Size (bp)**	**References**
**Primers used for cloning**			
E2e_fwd	CCCGAAGCTTGCACTTGGATTGCAAACCTGAATTC	~1,030 bp	(29)
E2e_rvs	CCCCGCTCGAGTGGACTCAGCGAAGTAATCCCG		
**Primers used for RNA BVDV detection in mice tissue samples**			
5UTRfwd	CTAGCCATGCCCTTAGTAGGACTA	~292 bp	(30)
STAR-Trev	CAACTCCATGTGCCATGTACAGCA		

Restriction sites for *Hind*III and *Xho*I enzymes are underlined.

### SDS-PAGE and Western blot

The expression of a recombinant protein named E2e was evaluated by SDS-PAGE and Western blot. Thus, the purified protein was diluted in sample loading buffer for SDS-PAGE and boiled at 100°C for 5 min. The recombinant protein was visualized in SDS-PAGE stained with Coomassie brilliant blue. Moreover, the recombinant protein was transferred onto a nitrocellulose membrane (Millipore Darmstadt, Germany) and probed with an anti-His tag monoclonal antibody (Invitrogen, Carlsbad, CA, USA), an anti-*myc* monoclonal antibody (Invitrogen, Carlsbad, CA, USA), or an anti-BVDV-E2 mouse monoclonal antibody (VMRD, Pullman, WA, USA), followed by a secondary horseradish peroxidase-conjugated anti-mouse IgG antibody (Sigma Aldrich, Saint Louis, MO, USA), and visualized by chemiluminescence (Thermo Fisher Scientific, Waltham, MA, USA).

### Vaccine preparation

Emulsification of recombinant E2e protein was prepared 1 day before immunization using mineral-oil-based water-in-oil adjuvant, commercially known as Montanide™ ISA 61 VG (SEPPIC, Paris, France), as previously described (31). The candidate vaccine was formulated by mixing purified recombinant E2e protein and Montanide™ ISA 61 VG to obtain a 50-ug final concentration of 500 ul per dose. Then, emulsification of negative controls using PSS + Montanide™ ISA 61 VG was performed.

### Animals, immunization, and viral challenge

The study was performed in 6–8-week-old specific pathogen-free BALB/c female mice (*n* = 6) obtained from the Biotechnology Institute-Universidad Nacional Autónoma de México (IBt-UNAM). All animals were maintained under pathogen-free conditions and handled in strict accordance with the guidelines and protocols approved by the Care and Use for Experimental Animals Sub-committee from the Facultad de Medicina Veterinaria y Zootecnia-Universidad Nacional Autónoma de México (FMVZ-UNAM). Mice had a 7-day acclimatization period before the onset of the trial. All vaccination and BVDV challenge procedures were performed in Animal Biosafety Laboratory Level 2 containments.

**Figure 1:**
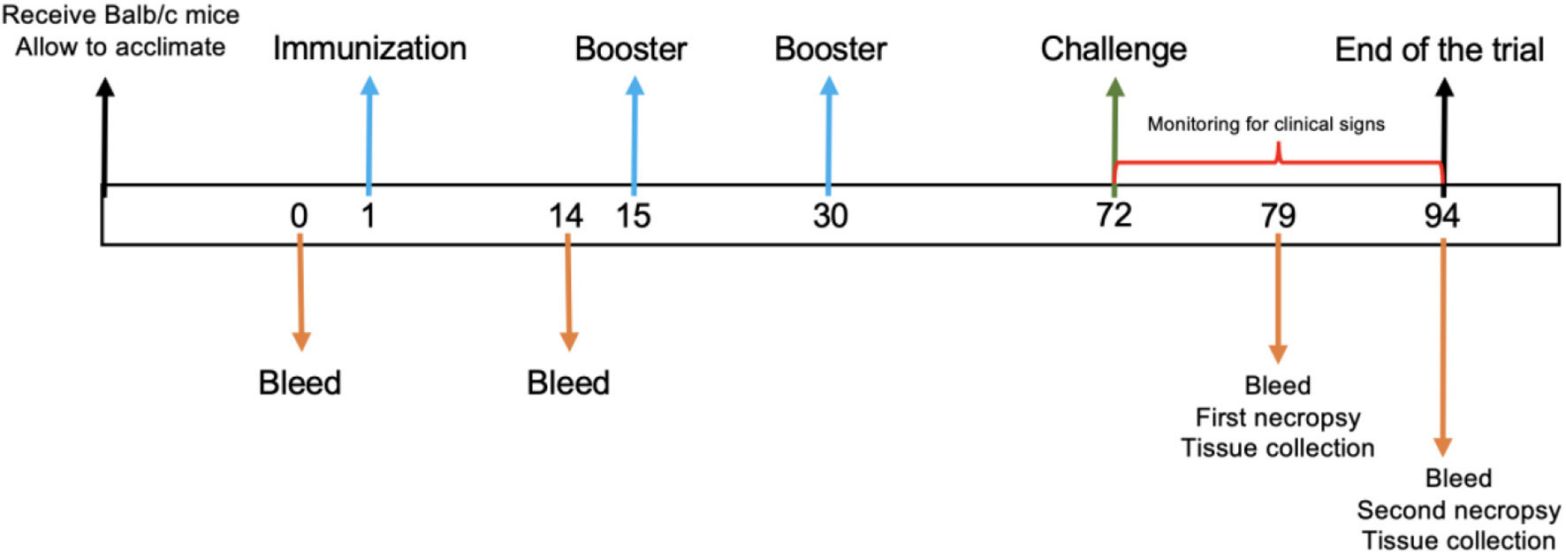
Experimental timeline. A schematic diagram of immunization protocol. The numbers indicate the days during the trial. Arrows indicate the trial procedure.

Following the acclimation period, six mice of 7–9 week old were randomly allocated to each of the five treatment groups and intraperitoneally immunized as follows: Group 1 was immunized three times with 50 μg of BVDV recombinant protein E2e; group 2 was immunized three times with 50 μg of BVDV recombinant protein E2e formulated in Montanide™ ISA 61 VG; group 4 was inoculated three times with physiological saline solution (PSS) emulsified with Montanide™ ISA 61 VG; and group 5 was inoculated with PSS and served as a negative control group. Immunization was performed on days 1, 15, and 30. Additionally, group 3 was included as a positive control for the BVDV challenge. Subsequently, 6 weeks after the third immunization, each mouse from groups 1–4 was challenged with 1 × 10^6.2^ TCID 50/ml of BVDV NADL strain (ATCC VR-534) administration through the orogastric route using a No. 8 straight stainless steel feeding cannula (Cadence Science, Inc. USA). On days 7 and 21 post-challenge (days 79 and 94 of the trial), half of the animals from each group were euthanized with CO_2_ gas to collect blood and tissue samples (Figure 1). After the viral challenge, mice were monitored for clinical signs daily.

### Sample collection

At given time points, mice were bled via retro-orbital sinus collecting 250–300 μl blood samples in 1.5 mL tubes without an anticoagulant. Serum was recovered from blood after centrifugation at 4,000 rpm for 10 min and preserved at −20°C until use. Serum from mice was collected on days 0, 1, 79, and 94 (Figure 1) for virus neutralization assay and RT-PCR. Furthermore, mice were euthanized on days 7 and 21 after the viral challenge, and the lung, stomach, liver, spleen, intestine, kidney, and brain were removed at necropsy for further analysis.

### Reverse transcriptase-polymerase chain reaction

Total RNA from lung, stomach, liver, spleen, intestine, kidney, and brain samples was extracted using TRIzol reagent according to the manufacturer's instructions. The RNA obtained from each sample was subjected to RT-PCR. The detection of BVDV in tissue samples by RT-PCR was performed using 5UTR/STAR primers as previously described in the study by Mahony et al. (30).

### Virus neutralizing titration

A standard virus neutralization test (VNT) was performed to detect antibodies against BVDV using the serum of mice collected on days 1 and 15 of the immunization trial and days 7 and 21 after the challenge. In brief, a serum pool from mice of each experimental group was submitted to VNT. Antibody neutralization titers were determined using the cytopathic viral strain BVDV-1a (ATCC VR-534) used in the challenge assay. Pool serums were inactivated at 56°C for 1 h and diluted with DMEM and run in triplicate using serial 2-fold dilutions from 1:4 to 1:32,768 in 96-well plates. In brief, 200 TCID_50_ of the viral strain was added to each well and incubated at 37°C in a humidified atmosphere of 5% CO_2_. After 1 h incubation, 2 × 10^4^ MDBK cells were included per well. Cell wells without the virus were used for every serum sample as negative controls. Plates were incubated for 96 h, and end points of antibody concentration were obtained by microscopic evaluation of the MDBK monolayer for the cytopathic effect. Endpoint titers were calculated using the Spearman–Kärber method (32). The titers were expressed as the reciprocal of the highest dilution that neutralized viral infectivity.

### Clinical evaluation

Clinical examinations were performed by a veterinarian daily after the BVDV challenge. In addition, the development of clinical signs was monitored during the post-challenge period.

### Histopathology and immunohistochemistry

Tissue samples were collected at necropsy from all animals surveyed in this study, including the lung, stomach, liver, spleen, intestine, kidneys, and brain. Tissues were fixed in 10% buffered formalin after at least 48–72 h. Paraffin-embedded blocks were sectioned in 4 μm, stained with hematoxylin and eosin, and examined for lesions by light microscopy. The histopathological findings in the mice after the BVDV challenge were evaluated by a pathologist who was blinded to the treatment regimen. A grading scoring system was utilized to assess histopathological lesions. The grading system was as follows: –/+ indicated incipient, + indicated mild, +/–++ indicated mild/moderate, ++ indicated moderate, ++/+++ indicated moderate/severe, and +++ indicated severe.

Fixed tissues were submitted to immunohistochemistry (IHC) for BVDV antigen detection. In the study, 4-μm thick paraffin-embedded tissue sections were deparaffinized and hydrated through graded alcohol series. The primary antibody was an IgG1 monoclonal antibody anti-BVDV named 3.12F1 (VRMD) diluted in a ratio of 1:2,000 in phosphate-buffered solution (PBS)-Tween 20 and used according to manufacturer's instructions. The secondary antibody used was biotinylated anti-multispecies (Invitrogen). Streptavidin-horseradish peroxidase and diaminobenzidine betazoid (DAB) were used to develop the color reaction. Subsequently, tissue sections were rinsed, counterstained, mounted, examined by light microscopy, and photographed. Tissue sections were incubated with PBS-Tween 20 instead of the primary antibody before treatment with secondary antibodies being used as negative controls. The BVDV immunohistochemical staining was graded and evaluated using a semiquantitative intensity scoring system: –: no detectable BVDV antigen; –/+: weak or faint BVDV antigen detection; +: minimal BVDV antigen detection; ++: moderate BVDV antigen detection ++/+++: moderate-to-intense BVDV antigen detection; and +++: intense BVDV antigen detection.

### Statistical analysis

The RT-PCR results were categorized using two-way clustering hierarchical cluster analysis (TWCHA) and a statistical method that classifies results into clusters based on their similarities (33). The results are organized in a matrix of *N x M* dimensions. N is the number of experimental groups, and *M* is the BVDV-positive percentage values obtained from tissues evaluated using the RT-PCR results. Data were robustly standardized, and the complete linkage method defined the distance metrics between clusters. The cluster analysis results are presented as dendrograms. The order of clusters is used to reorder the columns and rows of a heat map showing the percentage of BVDV-positive results by RT-PCR per tissue.

After immunization, the significance of the differences in neutralizing antibodies titer was estimated by Student's *t*-test performed in GraphPad software 6.0 version (GraphPad Prism, Software Inc., La Jolla, CA). The significant difference in tissue immunopositivity among the immunized and control groups was determined by a one-way analysis of variance (ANOVA) followed by Tukey's multiple comparison test performed in JMP software 11 version. Statistical significance was reported as follows, and a *p*-value of < 0.05 indicated a statistically significant difference.

## Results

### Expression and purification of BVDV E2 glycoprotein

**Figure 2:**
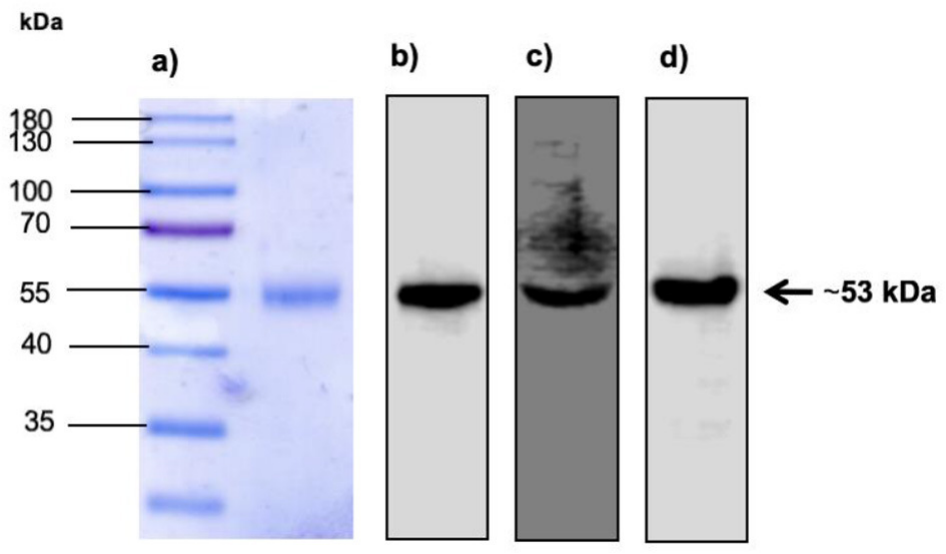
Expression of BVDV E2 recombinant protein in SDS-PAGE and Western blot. **(A)** Lane 1: Molecular weight marker. Lane 2: Expression of E2e recombinant protein recovered and purified from the supernatant of HEK-293T transfected cells. **(B)** Detection of E2e recombinant protein by Western blot using monoclonal anti-His tag antibodies diluted 1:1,000. **(C)** Detection of E2e recombinant protein by Western blot using anti-BVDV antibodies diluted 1:1,000. **(D)** Detection of E2e recombinant protein by Western blot using monoclonal anti-*myc* tag antibodies diluted 1:1,000.

We evaluated the expression and purification of BVDV recombinant E2e glycoprotein obtained from supernatants of transfected HEK-293T cells by Western blotting. Miscellaneous proteins were removed by purification. Correct protein expression and purification were confirmed by detecting a protein with a molecular mass of ~53 kDa, as shown in Figure 2.

### Clinical evaluation

No clinical signs of disease or behavior changes in mice utilized during this study were registered.

### RT-PCR from tissue samples and TWHCA

**Figure 3:**
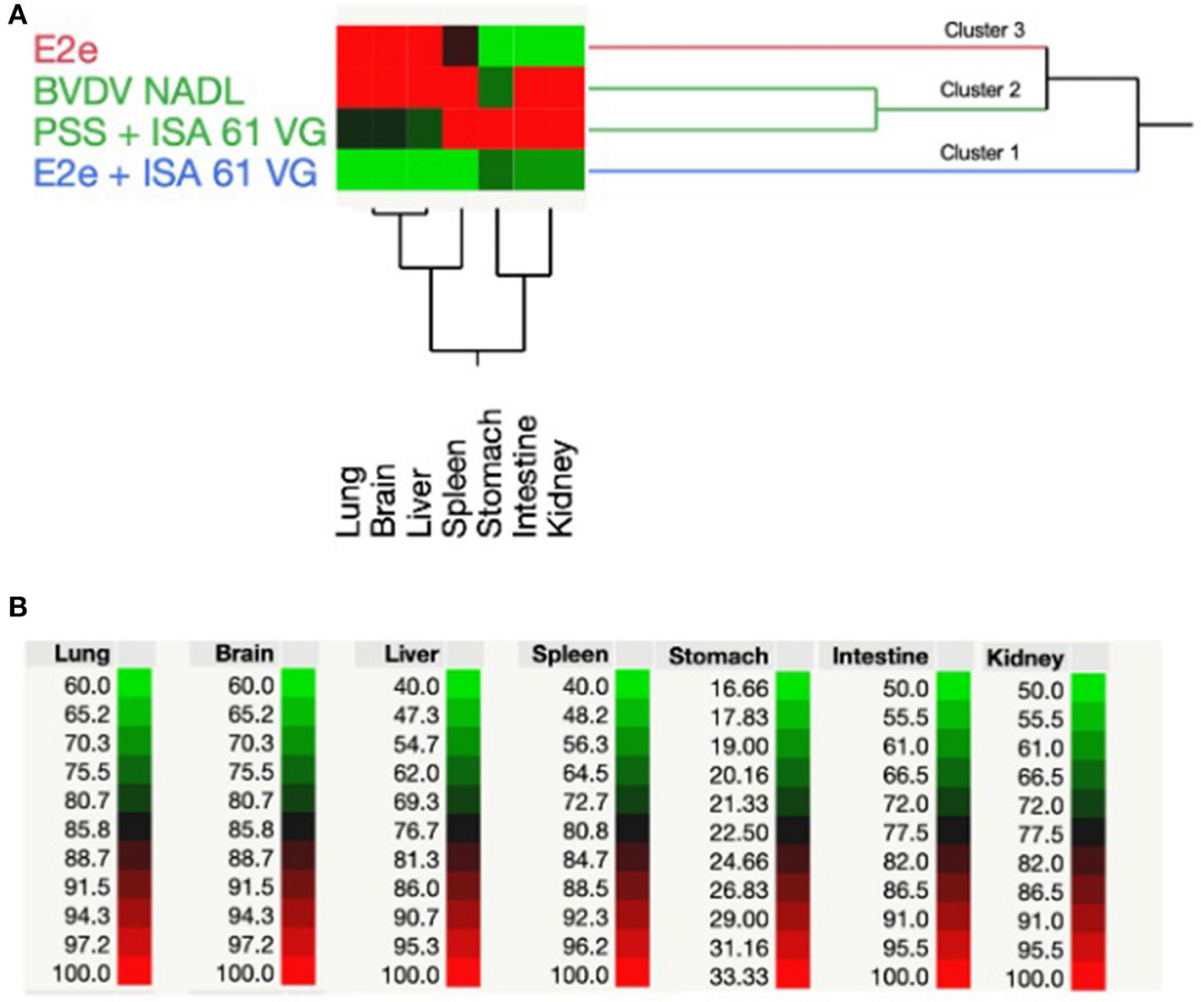
Two-way hierarchical cluster analysis based on positive RT-PCR results of BVDV detection. **(A)** Dendrogram shows the grouping pattern of BVDV RNA-positive detection in the lungs, brain, liver, spleen, stomach, intestine, and kidneys from experimental groups of mice used in this study. **(B)** Spectrum of colors between green and red shows the correlation intensity among the treatment of each experimental group and BVDV-positive percentage values obtained from mice tissues evaluated using RT-PCR results.

We conducted RT-PCR to detect BVDV RNA in tissue samples. Furthermore, BVDV-positive results from RT-PCR were analyzed using TWCHA resulting in the clustering of experimental groups based on their ability to harbor BVDV replication. Accordingly, three main clusters (1–3) were distinguished in the TWCHA analysis (Figure 3). Cluster 1 comprises only the experimental group 2 of mice immunized with E2e + ISA 61 VG. Groups 3 and 4, used as a positive control of BVDV challenge and immunization, were grouped within Cluster 2. Finally, Cluster 3 included experimental group 1 of mice immunized with solo E2e recombinant glycoprotein (Figure 3).

When comparing clusters, it was found that Cluster 2 had the highest average percentage of BVDV detection through RT-PCR in most of the examined tissues. Therefore, Cluster 2 was associated with a higher BVDV challenge strain replication. Conversely, immunized mice with solo E2e recombinant glycoprotein and E2e + ISA 61 VG, mice experimental groups 1 and 2, respectively, showed the lowest percentage of BVDV detection in evaluated tissues. Interestingly, compared to Cluster 3, Cluster 1 showed lower BVDV-positive results, suggesting that mice immunized with E2e + ISA 61 VG formulation helped to reduce the BVDV replication in the mentioned tissues by the elicited immune response.

### Virus neutralizing titration

**Figure 4:**
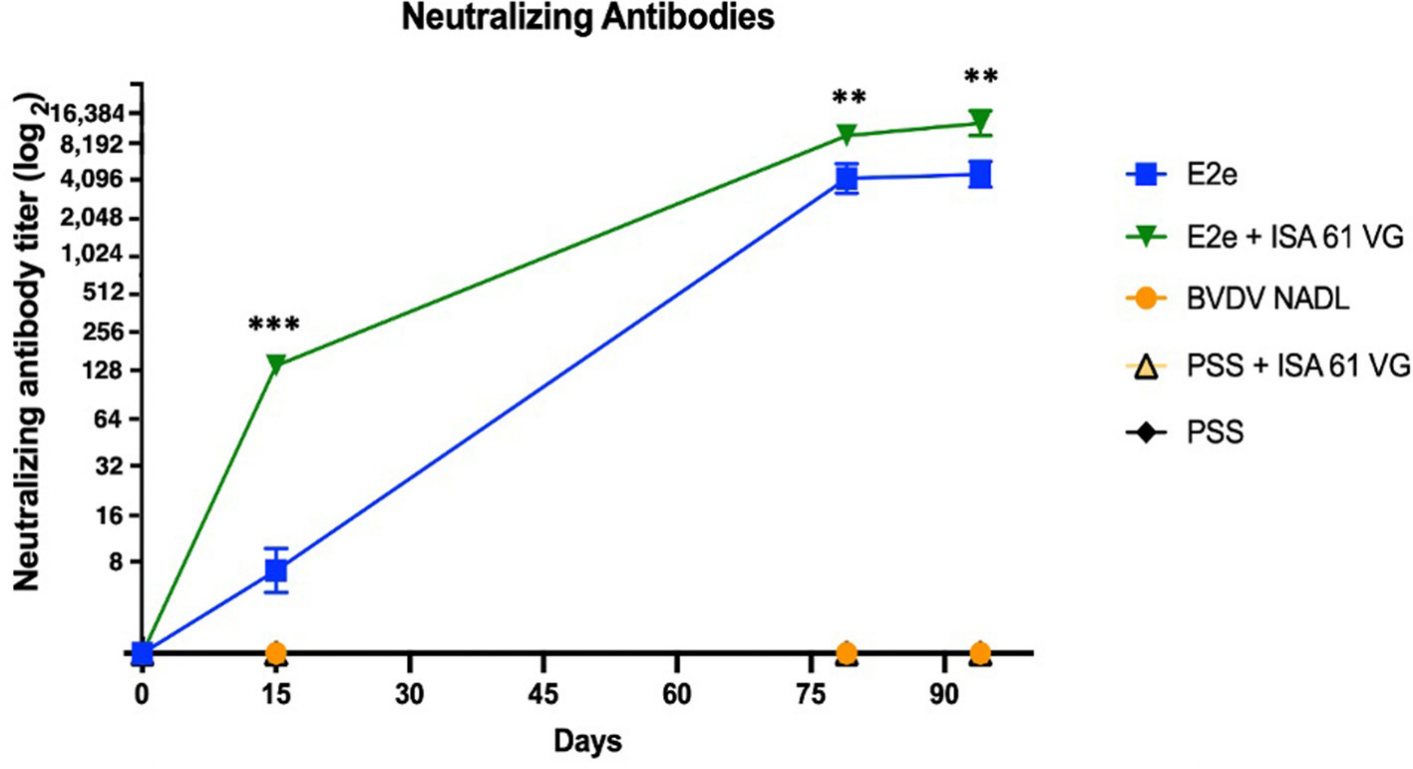
Neutralizing antibody titer. Comparison of viral neutralization titer of each experimental group. Neutralizing antibodies were detected by VNT. Titers are expressed as the reciprocal of the highest dilution that neutralized viral infectivity. ***p < 0.001, **p < 0.01.

To evaluate humoral response after vaccination, we measured the concentration of BVDV-neutralizing antibodies in the sera of challenged mice. Vaccinated mice from groups 1 and 2 were seroconverted after the first treatment inoculation. Conversely, mice from groups 3–5 remained seronegative until the end of the study. Neutralizing BVDV-specific antibodies were detected in serum collected during the three sampling periods on days 15, 79, and 94 post-immunization. Interestingly, obvious differences in antibody levels were observed between groups after the first immunization (Figure 4). Notably, the antibody levels continued to increase in the immunized groups throughout the experiment. For example, 2 weeks after the first immunization, the E2e and the E2e + ISA 61 VG groups displayed VN titers ranging from 8 to 16 and 8 to 128, respectively; therefore, these experimental groups had higher antibody levels than the control groups 3 and 4 (*p* < 0.05, and *p* < 0.001, respectively).

Moreover, the neutralizing activity of sera from group 2 was estimated to be 8,192 at 7 days after the viral challenge and up to 16,384 at 21 days after the viral challenge. Thus, VN titers were significantly higher in group 2 than in group 1 (*p* < 0.01), which induced VN titers between 2,048 and 4,096 in those sampling periods. Notably, although control groups 3 and 4 did not elicit any detectable neutralizing activity throughout the immunization trial, BVDV viral challenge might imply the increase of VNT titer in groups 1 and 2.

Both immunized groups developed a stronger neutralizing humoral response than the positive control group 4 (*p* < 0.001). Notably, the vaccination using E2e + ISA 61 VG elicited a significantly higher neutralizing antibodies level in a short period than group 1 (*p* < 0.001). No significant differences were found between groups 3 and 4. Data analysis revealed a significantly stronger neutralization activity in groups 1 and 2 compared with the BVDV NADL control group. No viral neutralization activity was detected in the negative control group.

### Histopathology and immunohistochemistry

To evaluate immune protection against BVDV challenge induced by the immunization protocol in this study's four experimental groups, histopathological examinations were performed in the lungs, stomach, liver, spleen, intestine, kidneys, and brain at 7 and 21 days post-challenge. All animals challenged with the BVDV strain showed mild-to-severe histopathological lesions, whereas mock-infected mice showed no lesions. The histopathological lesions found in mice after viral challenge are described. The lungs of mice inoculated with the BVDV reference strain exhibited mild-to-severe interstitial pneumonia and mild hyperplasia of bronchus-associated lymphoid tissue (BALT). Similarly, the lungs of PPS + ISA 61 showed moderate-to-severe interstitial pneumonia. Conversely, group 2 exhibited no severe pathological changes, whereas group 1 had moderate interstitial pneumonia.

**Figure 5:**
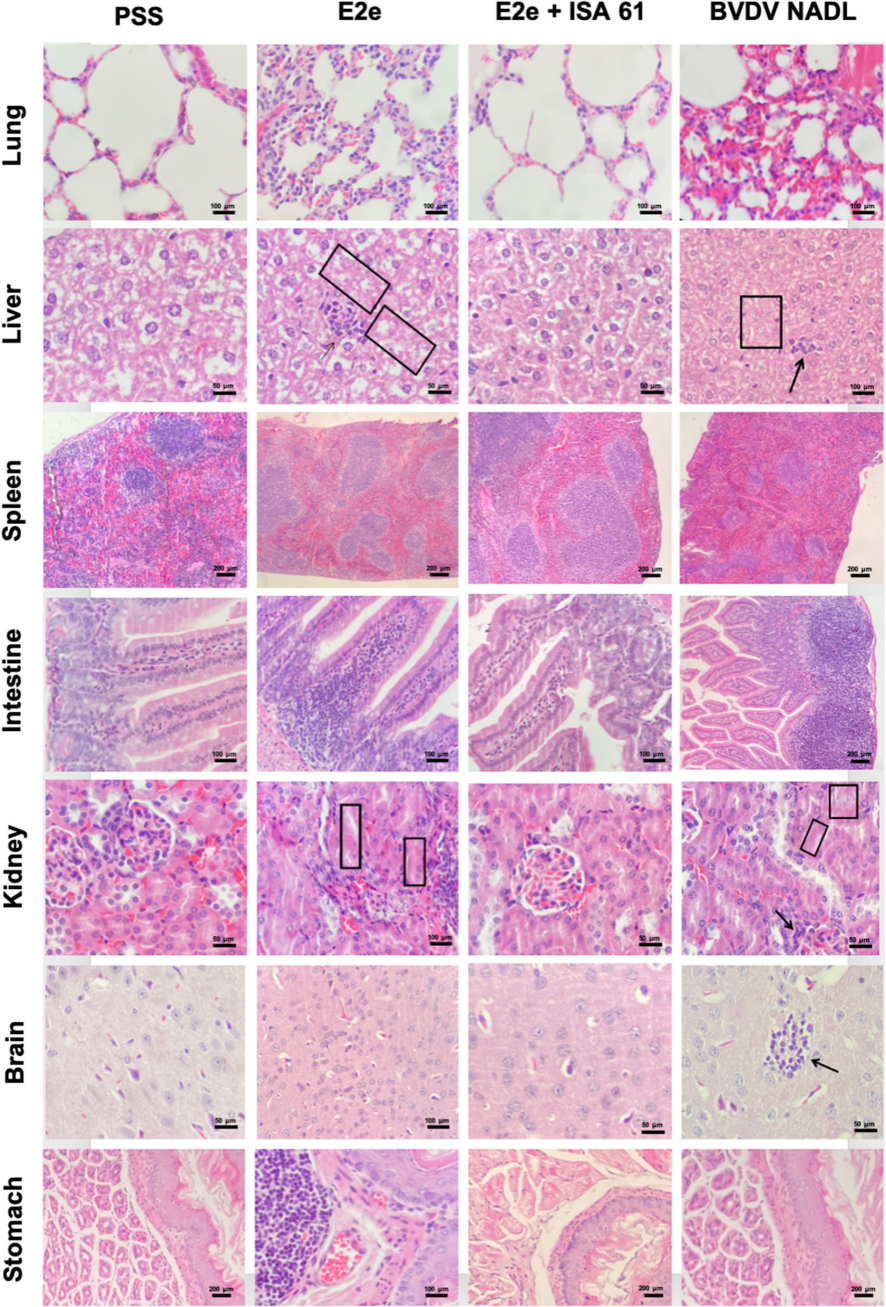
Representative histopathological features of the lung, liver, spleen, intestine, kidney, brain, and stomach sections of immunized and challenged mice from experimental groups 1–3. Group 5 (PSS) was included as a negative control and Group 3 as a positive control. Compared to the negative control group 5, mice immunized with solo E2e formulation and BVDV NADL strain showed coagulative necrosis and lymphocytic infiltration in the liver (squares and black arrows in liver tissue, respectively). Similarly, mice from groups 1 and 3 exhibited mild-to-moderate glomerulitis and tubular necrosis (black arrows and squares in kidney tissues, respectively).

The intestine of mice inoculated with BVDV reference strain and PPS + ISA 61 displayed more severe lymphoid infiltration than those of the immunized groups. The livers of BVDV reference strain-infected mice showed moderate coagulative necrosis. In contrast, only one mouse of the E2e + ISA 61 group showed mild coagulative necrosis, while the livers from immunized mice with E2e displayed mild-to-moderate coagulative necrosis in livers. Moreover, mild-to-moderate lymphocytic infiltration was also recorded (Figure 5). In addition, mild-to-moderate encephalitis and neural necrosis were noted in the positive control groups; however, in mice immunized with E2e, no brain lesions were observed, while incipient to mild encephalitis and neural necrosis were observed in one mouse from group 3 at 21 days post-challenge. Mild-to-moderate lymphoid depletion was observed in the spleens from positive control groups, similar to those from mice immunized with E2e; conversely, only mild lymphoid depletion was noted from the E2e + ISA 61 group.

The kidneys of the two positive control groups exhibited mild-to-moderate nephritis, glomerulitis, and tubular necrosis. Mice immunized with E2e showed mild-to-moderate nephritis, glomerulitis, and tubular necrosis, whereas, in mice immunized with E2e + ISA 61, no lesions were found in the kidneys. Furthermore, moderate hyperplasia gut-associated lymphoid tissue (GALT) was observed in the stomachs of mice inoculated with PSS + ISA 61. This lesion was also found in the stomachs of mice in the E2e group, while no lesions were found in the stomachs of mice in the E2e + ISA 61 group (Figure 5).

**Figure 6:**
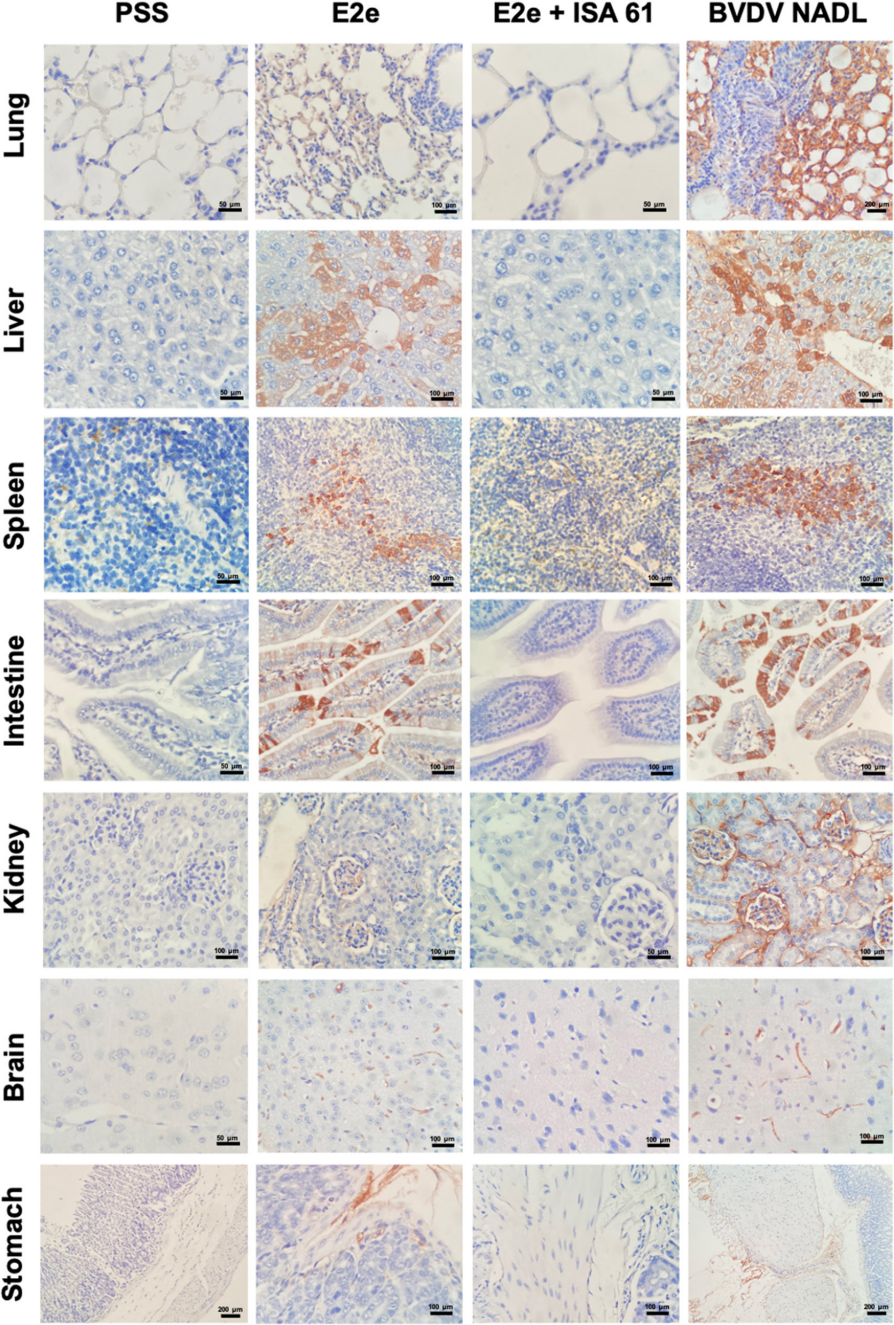
Immunohistochemical staining of BVDV in tissues from immunized and challenged mice. BVDV antigen was detected in the lungs, liver, spleen, intestine, kidneys, brain, and stomach of mice with variable immunopositivity in the experimental groups 1–3, whereas group 5 (PSS) was included as a negative control.

To assess the immune protective effects of E2e and the E2e + ISA 61 candidates, we performed immunohistochemistry (IHC) to evaluate BVDV antigen burden in mentioned tissues. Positive staining for BVDV antigen was detected in every evaluated tissue in mice from the BVDV NADL and PSS + ISA 61 groups. No BVDV antigen was detected in the negative control group. Significant differences were observed in the lungs, liver, and brain between the experimental groups. BVDV antigen was prominently less detected in lungs from the E2e + ISA 61 group in comparison with the E2e and PSS + ISA 61 groups. Moreover, an increased immunopositivity was detected in livers from the E2e, BVDV NADL, and PSS + ISA 61 groups in comparison with E2e + ISA 61 group. Similarly, more intense staining was noted in E2e, BVDV NADL, and PSS + ISA 61 group brain tissues, whereas, in the E2e + ISA 61 group, only one mouse displayed minimal BVDV antigen detection (Figure 6).

## Discussion

BVDV is prevalent worldwide and is responsible for a complex combination of clinical manifestations and immunosuppression. Additionally, BVDV infection of pregnant cattle with BVDV can cause abortions, stillbirths, and the birth of BVDV immunotolerant animals named persistently infected animals (PI) (34). Therefore, BVDV infection is a cause of substantial economic loss to the cattle industry (1). Currently, preventive measures such as BVDV vaccination have failed to confer broad protection against BVDV infections; hence, BVDV prevalence has not been reduced (35). Additionally, despite the availability of conventional BVDV vaccines, unwanted effects, when used improperly, may occur, such as reproductive disorders such as *in utero* infections, abortion, and stillbirths in pregnant cattle, recombination with field strains, development of mucosal disease presentation in PI animals, and immunosuppression (36–38). Owing to the significant risks of BVDV vaccine application to susceptible animals, developing improved, efficacious, and safe vaccine candidates is vital to prevent its occurrence and transmission. The BVDV E2 glycoprotein is highly immunogenic, containing T-cell epitopes within the three antigenic domains (39, 40); hence, it is described as the immune-dominant viral envelope protein containing neutralizing epitopes (41). Thus, neutralizing antibodies and T-cell response directed against E2 glycoprotein can confer protection (42, 43).

Recently, the development of several vaccine formulations using the E2 gene or E2 glycoprotein has been reported. The efficacy of the E2 recombinant glycoprotein used as subunit vaccines in several formulations is described. These formulations include the emulsification with novel adjuvant platforms, with preparations combining the E2 recombinant glycoprotein with inactivated BVDV, a combination of E2 recombinant glycoprotein with DNA vaccine encoding E2 glycoprotein, virus-like particles expressing the E2 glycoprotein. C-terminally truncated version of E2 recombinant glycoprotein was used as a subunit vaccine. The efficacy of these BVDV vaccine candidates has been evaluated in animal models such as goats, guinea pigs, cattle, and mice models with promising results. Depending on the study, the protection conferred against BVDV infection was evidenced by a reduction in clinical signs such as lymphopenia and lack of pyrexia, reduction in viral shedding, antibody neutralization titers, and an increase in CD4^+^ and CD8^+^ lymphocytes along with the cytokine levels for humoral and cellular immune responses (12, 44–49).

The murine model has been used to study the pathogenesis of other bovine diseases and antimicrobial and immunomodulatory agent activities to control bovine diseases (50, 51). Additionally, previous studies performed by Seong et al. (18) described histopathological lesions such as atrophy of the glomerulus, thickening of the alveolar wall, lymphocyte necrosis within the lymphatic nodule, and lymphocyte depletion in the spleen, in addition to lymphopenia, leukopenia, and thrombocytopenia after BVDV CP infection (18, 19). Similarly, acute BVDV infection in cattle is characterized by interstitial pneumonia with alveolar septal thickening, lymphocyte depletion in lymph nodes, glomerulonephritis, and lymphopenia (22–24). Therefore, the resemblance in alterations and tissue lesions caused by the BVDV infection makes the murine model suitable for the preliminary evaluation of vaccine candidates.

In this study, we evaluated the humoral immune response and protection against BVDV infection in mice, conferred by two formulations of candidate vaccines that included BVDV recombinant E2 protein (E2e) alone and E2e with Montanide ISA 61 VG adjuvant. We found that immunization with E2e + ISA 61 elicited a stronger immune response and greater protection against BVDV than solo E2e immunized mice.

The results obtained by RT-PCR indicated that BVDV RNA could be easily detected in most mice tissues from the BVDV NADL and PSS + ISA 61 groups. In addition, these groups showed a higher percentage of BVDV positivity. On the contrary, the results from the E2e and E2e + ISA 61 groups evidenced fewer BVDV RNA detection, with E2e + ISA 61 being the group with a higher percentage of BVDV negativity demonstrated. The low detection level may be attributable to a reduced BVDV ability to infect, replicate, and distribute in target tissues in mice after the BVDV challenge was associated with using the described vaccine candidate E2e + ISA 61.

Evaluating neutralizing antibody responses elicited by immunization is an important criterion to determine vaccine efficacy. Previous studies on immunized cattle have suggested that a minimum titer of 216 is required to avoid the development of severe clinical BVD manifested by fever, leukopenia, thrombocytopenia, and diarrhea (52). Conversely, studies performed by Beer et al. indicated that antibody titers > 512 indicate protection against BVDV infection evidenced by the lack of leukopenia and no viral isolation from challenged cattle (53). Seroconversion was detected in both immunized groups after the first immunization; notably, VN titers continued to rise in these groups and were maintained throughout the experimental period. After the first immunization, we detected significant differences in antibody titers of the immunized groups due to the humoral response elicited by the E2e group being weaker than that induced by the E2e + ISA 61 group. Noteworthy, following booster, the VN titers 7 and 21 post-challenge remain higher in the E2e + ISA 61 group than the response stimulated in the E2e group. Thus, overall, the immunization protocol with the E2e + ISA 61 group induced an early and higher humoral response, sufficient to afford protection. The latter is suggested by the reduced BVDV RNA and antigen detection in evaluated tissues since the antibody levels are likely to correlate positively with the level of protection (15). Interestingly, no antibody-neutralizing activity was detected in BVDV NADL and PSS + ISA 61 groups. This is consistent with the results obtained by Ren et al., where no detection of antibodies was evidenced 28 days post-inoculation with the BVDV NADL reference strain (54). In contrast, immunized cattle can display high BVDV-neutralizing antibodies titers starting at 15 days post-BVDV inoculation (55).

Subsequently, the protective effects of vaccine candidates were analyzed by assessing histopathology changes and BVDV antigen detection in mice tissues after the viral challenge. The lungs, liver, and brain of mice in the E2e, E2e + ISA 61, BVDV NADL, and PSS + ISA 61 groups were damaged to different degrees after the BVDV challenge. However, the E2e + ISA 61 group exhibited minor damage between groups. Similarly, this group elicited a substantially reduced degree of BVDV antigen detection compared to the other groups. These results are consistent with those obtained in RT-PCR and VNT, indicating that using E2e + ISA 61 formulation as a vaccine candidate limited BVDV replication in mice.

Conversely, immunopositivity and a higher level of lesions in tissues suggested increased BVDV replication; therefore, decreased protection was induced when E2e was used. Similarly, immunization of mice with recombinant E2e protein elicited higher VN titers; however, the developed humoral immune response did not confer protection after the BVDV challenge. This was supported by BVDV antigen detection and histopathology examination, where no significant difference was observed compared to those obtained in the positive control groups.

Several studies based on vaccination with E2 glycoprotein expressed in different systems have been previously described (44, 56–58); however, the use of emulsified BVDV E2 glycoprotein has not yet been described in the mice model. Moreover, our findings showed that, using adjuvants, such as ISA 61 VG, confers enhanced efficacy in immune response; thus, this constitutes an interesting alternative with great potential for use in BVD control. In addition, using this vaccine candidate could prove to be a valuable tool in BVDV control programs to differentiate between vaccinated and infected animals (59).

However, one of the limitations of our study was the lack of characterization of cellular immune response in immunized mice or detection of molecular markers to define the immune response profile elicited by the formulations used in this study. In addition, other immune protection indicators, such as viremia, viral shedding, lymphopenia, and thrombocytopenia, should be considered for further analyses.

In summary, our results indicated that emulsification of E2e protein with ISA 61 adjuvant and boost strategy promotes a strong humoral immunity, which provided protection against BVDV challenge in mice than solo E2e immunization. Therefore, E2e + ISA 61 formulation represents a viable vaccine candidate for the target species. However, studies on cattle need to be done to evaluate their safety and efficacy and further application in BVDV control strategies. Additionally, a comparison and correlation of the induced immune response in mice and bovines using the candidate subunit vaccine described here need to be performed.

## Data availability statement

The original contributions presented in the study are included in the article/supplementary material, further inquiries can be directed to the corresponding author.

## Ethics statement

The animal study was reviewed and approved by the Care and Use for Experimental Animals Sub-committee (SICUAE) from the Faculty of Veterinary Medicine of the National Autonomous University of Mexico (FMVZ-UNAM).

## Author contributions

NG-R, FB-A, AV-R, CA, and SL participated in the study design, supervision, and analysis. NG-R performed the experiments and wrote the manuscript. NG-R, LV-M, and CC-P performed the immunohistochemical assay. LV-M and CC-P performed histopathological and immunohistochemical analyses. Funding acquisition was obtained by FB-A, CA, and SL. All authors contributed to the article and approved the submitted version.
